# *Echinococcus multilocularis* specific antibody, systemic cytokine, and chemokine levels, as well as antigen-specific cellular responses in patients with progressive, stable, and cured alveolar echinococcosis: A 10-year follow-up

**DOI:** 10.1371/journal.pntd.0010099

**Published:** 2022-02-02

**Authors:** Beate Grüner, Lynn Peters, Andreas Hillenbrand, Patrick Voßberg, Jonas Schweiker, Elisabeth G. Rollmann, Laura H. Rodriguez, Jasmin Blumhardt, Sanne Burkert, Peter Kern, Carsten Köhler, Peter T. Soboslay

**Affiliations:** 1 University Hospital of Ulm, Department of Internal Medicine III, Division of Infectious Diseases, Ulm, Germany; 2 Department of General and Visceral Surgery, Ulm University Hospital, Ulm, Germany; 3 University Clinics Tübingen, Institute for Tropical Medicine, Eberhard-Karls University, Tübingen, Germany; National Institute of Allergy and Infectious Diseases, UNITED STATES

## Abstract

**Background:**

The infestation with *Echinococcus multilocularis* larvae may persist in humans for up to decades without evident clinical symptoms. Longitudinal investigations are needed to understand the dynamic immunological processes in alveolar echinococcosis (AE) patients associated with an active and progressive, a stable or a regressive course of disease.

**Methodology/Principal findings:**

This study evaluated the *E*. *multilocularis* specific antibody responses, systemic cytokine, and chemokine serum levels over a 10-year follow-up period, as well as cellular responsiveness in AE patients. Our results demonstrate a rapid decrease in antibodies against *E*. *multilocularis* specific antigen Em2+. Especially in cured patients, these antibodies remained negative, making them a significant predictor for cured AE. *E*. *multilocularis* specific IgG4, and indirect hemagglutination IHA decreased later in time, after around 5 years. While total IgE did not show significant dynamics over the course of disease, *E*. *multilocularis* specific IgE decreased after one to two years, and increasing levels were a significant predictor of progressive disease. There was no significant change in systemic IL-8, IL-9, CCL18 or CCL20 serum levels over time. Univariate analysis across groups indicated lower IL-8 levels in cured patients; however, this result could not be confirmed by multivariate analysis. Levels of CCL17 decreased during treatment, especially in cured patients, and thus might serve as a predictive or risk factor for progressive disease. Levels of IL-10 and CCL13 decreased during disease, especially after five and ten years of intervention. The *E*. *multilocularis* antigen (EmAg) inducible cellular productions of MCP1(CCL13), TARC(CCL17) and PARC(CCL18) were lowest in patients with cured AE and infection-free controls, while the EmAg inducible cellular production of IFN-γ increased after cure. Significant positive cytokine and chemokine correlations were observed in AE patients for IL-9, IL-10, CCL13(MCP-4), CCL17(TARC) and CCL20(LARC)(for all p<0.001). *E*. *multilocularis* specific IgG4 response correlated positively with TARC (p<0.001). Both markers enhanced over time in progressive disease and decreased after cure. The levels of IL-8, IL-10, MCP4 and LARC enhanced with AE regression.

**Conclusions/Significance:**

Repeated biomarker surveys are advisable to evaluate progression or regression of disease during longitudinal follow-up and such analyses can support imaging techniques and improve staging of AE patients.

## Author summary

Alveolar echinococcosis (AE) is a severe disease caused by *Echinococcus multilocularis*, the fox tapeworm. Humans exposed to *E*. *multilocularis* may develop severe AE with progressive tissue and organ infiltrating growth of the larval stage. The *E*. *multilocularis* larvae appear to have developed effective immune evasion mechanisms which facilitate an asymptomatic incubation and an extended host and parasite coexistence for decades. Over a 10-year follow-up, this investigation aimed to gain a better understanding of the immunological process associated with an active and progressive, a stable or a regressive course of AE. In summary, the rapid decrease of antibodies against the *E*. *multilocularis* specific antigen Em2+, especially in cured patients, makes them a significant predictor for cured AE. The positive relation of *E*. *multilocularis* specific IgG4 responses and chemokine levels of TARC can indicate AE progression when both enhance over time. Enhanced levels of cytokines IL-8, IL-10, and chemokines MCP4 and LARC may predict AE regression. Repeated biomarker surveys are advisable to evaluate progression or regression of AE during longitudinal follow up, and such analyses can support imaging techniques and improve staging of AE patients.

## Introduction

Alveolar echinococcosis (AE) is a severe disease caused by the cestode *Echinococcus multilocularis*, the fox tapeworm. The disease is most prominent in northwest China but also occurs in central Europe [1]. AE morbidity and treatment costs are high, with a lethal course if left untreated [2–5]. Together with cystic echinococcosis, AE is considered a neglected disease [6,7]. Moreover, it is an emerging disease, and an increase in human cases of AE in Europe have been observed over the past decades [2,8–10].

Humans exposed to *E*. *multilocularis* may develop severe AE with progressive tissue and organ infiltrating growth of the larval stage [11]. *E*. *multilocularis* larvae may massively proliferate in liver and other organs over decades without evident clinical symptoms, and in this process the evasion and regulatory mechanisms of the parasite play a crucial role on the human host immune response [12–14]. The *E*. *multilocularis* larvae appear to have developed effective immune evasion mechanisms which facilitate an asymptomatic incubation and an extended host and parasite coexistence. The larvae may persist for decades exerting a progressive tissue invasive tumour-like growth [1,2,15], but few AE patients may present with a healing course of the disease. This diversity suggested immunological mechanisms which may control the course of AE. Characteristic for a certain resistance to AE is the occurrence of Th1 type cytokines, while elevated production of IL-10 and Th2 cytokines were associated with progressive Em larval growth [16–18].

Only in 10–30% of *E*. *multilocularis* seropositive cases [11] clinical disease will develop, suggesting that effective immune responses may have eliminated the larvae when penetrating the intestinal wall at an early stage of infestation [19]. Patients who express Th1-type immune response are more likely able to limit or regress larval growth by forming peri-parasitic granulomas with macrophages and T cells, as well as lesions, or fibrosis and necrosis to encapsulate the parasite [20,21]. Successful immune evasion of the parasite leads to a tolerant Th2 immune response which is unable to prevent the *E*. *multilocularis* larval growth [15,22]. The disease spectrum is dependent on an acquired deviation of Th1-related immunity, and the spontaneous secretion of IL-10 by the PBMC was identified as the immunological hallmark of patients with progressive forms of AE involved in the maintenance of tolerance and persistence of the parasite [23–25]. Reactivity of peripheral blood cells to E. multilocularis antigens persisted for years in AE patients after complete resection of the parasitic lesions suggesting that residual parasite tissues will continue to stimulate cellular responses [26]. *E*. *multilocularis* may expand regulatory T cell responses [27] and promote a largely Th2-biased response which allows long-term parasite survival, proliferation, and maturation [17,21]. However, only few studies have longitudinally addressed the changes of immunity at different stages of AE in patients.

This investigation aimed to gain a better understanding of the immunological processes at distinct stages of AE disease. Over a 10-year follow-up, antibody responses to *E*. *multilocularis* and systemic cytokine and chemokine levels, as well as parasite antigen-specific cellular responses were assessed in patients with progressive, stable, and cured AE. A better understanding of the differences in the elicited immune response may reveal new treatment rationales.

## Material and methods

### Ethics statement

All patients gave their written consent to participate in this study, and approval for the latter was obtained from the ethical board at University Clinics of Ulm (Ethik-Kommision Antrag Nr. 71/2004, 71/2004-Amendment2010 and Antrag Nr. 372/2015).

### Alveolar echinococcosis (AE) patients and disease staging

All AE patients were admitted to and consulted at the Echinococcosis Reference Centre at University Hospitals of Ulm, Germany. The Echinococcosis Centre in Ulm and the University Clinics of Tübingen are in the southwest of the federal state of Baden-Württemberg in Germany, both towns being 70 km apart, in an endemic area for *E*. *multilocularis* [25]. The AE patients in the present study were classified into cured, stable, and progressive according to established criteria [3] at the time of the study.

Diagnosis and classification of AE was performed using positive imaging, serology and histology and AE-case-definition according to criteria, which were established by the WHO Informal Working Group on Echinococcosis [28] as well as PNM–staging system (P = parasitic mass in the liver, N = involvement of neighbouring organs, and M = metastasis) [1–3,29], and most patients were re-examined regularly during follow-ups.

The AE patients were staged into progressive, stable and cured AE. The application of imaging techniques for AE staging is essential. Ultrasound (US) and computed tomography (CT) were used to confirm alveolar echinococcosis [1], and magnetic resonance imaging (MRI) can provide a more differentiated diagnosis, to identify blood vessel structures before surgery [3,30]. Positron emission tomography (PET) or combined PET-CT was used to shows areas of high metabolic activity, which may indicate inflammatory reactions and possible activity of the parasite [31].

Clinical status was classified as:

1. cured: patients who underwent curative surgery and showed no signs of residual parasitic mass or relapse for at least two years postoperative.
2. stable disease without BMZ treatment: inoperable patients without benzimidazole treatment who show no signs of progressive disease
3. stable disease with BMZ treatment: inoperable patients on continuous benzimidazole treatment who show no signs of progressive disease
4. progressive disease: inoperable patients with growing parasitic lesion and/or associated biliary or vascular complications despite benzimidazole treatment.

### Serum collection from AE patients and healthy controls

Serum samples from AE patients were collected at three-time points; in 2005 (year 1, n = 101 samples), 2010 (year 5, n = 57 samples) and 2016 (year10, n = 59 samples). Infection-free healthy controls were blood donors (n = 35) from University Clinics of Tübingen; all controls were negative in *E*. *multilocularis* antigen-specific antibody ELISA responses. For this study, only patients that had donated several times were included to enable cross-time analysis.

### *Echinococcus multilocularis* antigen (EmAg) specific IgG responses

Antibody-screening was performed with the Echinococcus-specific IgG indirect hemagglutinin test (IHA) and confirmatory testing was done by the commercialized *E*. *multilocularis*-specific Em2+-antigen ELISA as described previously [32]. The detection of EmAg-specific IgG1, IgG3, IgG4 and IgE was applied as described previously [33,34].

For analyses of EmAg-specific IgG sublass responses, ELISA microtiter 96 well plates (CORNING; EIA/2 CatNo.3690) were coated overnight at 4° C with *E*. *multilocularis* metacestode larval extract (EmAg, 12 μg/ml) in phosphate-buffered saline (PBS). For *E*. *multilocularis* antigen (EmAg) preparation, solid metacestode tissues were isolated from metacestode-infected mongolian gerbils (*Meriones unguiculatus*), tissues were washed in phosphate-buffered saline (PBS, pH 7.2–7.4) and ground on ice in a Ten-Broek tissue grinder until a homogenous liquid extract appeared. The antigen suspension was then sonicated twice on ice for 10 min (30% cycle, Model 250, Branson Ultrasonics, Danbury, USA). The liquid extract was then collected and centrifuged at 15,000 *g* for 30 min at 4° C. The supernatant was sterile-filtered (0.22 μm) and stored in aliquots at -70° C for further use. The protein concentration of these *E*. *multilocularis* antigens was determined using the bicinchoninic acid protein determination kit (Pierece, Rockford, IL, USA). After coating, ELISA plates were washed twice with PBS 0.05% Tween 20 (PBST, pH 7.4) and subsequently blocked with PBS containing 5% BSA for 1.5 hours at 37° C. For the analyses of antibody responses IgG1, IgG3 and IgG4 were selected because the carbohydrate rich antigens located on the laminated layer of metacestode larvae will trigger these IgG subclasses. Serum samples from AE patients and echinococcosis-free controls were diluted 1:10 with 5% BSA in PBS, added to each well and incubated at 37° C for 2 hours. Then plates were washed three times and detection was performed with monoclonal anti-human IgG_1_, IgG_3_, or IgG_4_ antibody conjugated with HRP (Thermo Fisher, A-10648, A-10654, 05–3600) diluted 1:500 with 5% BSA in PBS and added for 1.5 hours at 37° C. After three times washing, Tetramethylbenzidine (TMB) solution was added, and colour reaction was stopped with 0.5 M sulfuric acid. Optical density of sample wells was determined at 450 nm using the EL808 Photometer (BIOTEK) and Gen5 1.11 software. The data were processed and statistically evaluated with JMP 14.2.0.

### Isolation of PBMC, cell culture experiments and determination of cytokine and chemokine production

Peripheral mononuclear blood cells (PBMC) from AE patients were isolated by Ficoll gradient centrifugation and cultured *in vitro* as previously described [35]. Briefly, PBMC were adjusted to 1x10^7^/ml in RPMI supplemented with 25 mM HEPES buffer, 100 U/ml penicillin, 100 mg/ml streptomycin and 0.25 mg/ml amphotericin B. Freshly isolated PBMC were cultured at 2.5x10^6^ PBMC/ml in RPMI (as above) and stimulated with *E*. *multilocularis* metacestode antigen extract (EmAg, 50 μg/ml) or remained without stimulation (baseline) for 48h in 5% CO_2_ at 37° C and saturated humidity. Cell culture supernatants were collected after 48h and stored below –70° C until further use. Cytokine and chemokine secretion by stimulated PBMC was quantified by sandwich enzyme-linked immunosorbent assay (ELISA) using monoclonal and polyclonal antibodies for interleukin(IL) - 8 (IL-8, CXCL8), IL-9, IL-10, interferon-gamma (IFN-γ), and the CC chemokines MCP-1 (CCL2), MCP-3 (CCL7), MCP-4 (CCL13), TARC (CCL17), PARC (CCL18) and LARC (CCL20) as recommended by the manufacturers and described previously [34–36]. The detection limits of the cytokine and chemokine ELISAs (DuoSet, R&D Systems, Minneapolis, USA) were at 50 pg/ml.

### Data analysis

Statistical analysis was performed using IBM SPSS version 25. All variables were described with mean, median and standard deviation. Prior to every calculation, variables were tested for normal distribution using graphical methods such as histogram, P-P- and Q-Q-plot as well the Shapiro-Wilk test. Skewed data was log-transformed for further analysis. To assess changes in serological markers and cytokine levels over time, repeated-measures ANOVA, and paired t-test for normally distributed or Wilcoxon signed-rank test for non-parametric data were used. Comparisons across groups were conducted with ANOVA and independent t-test for normally distributed, continuous data and Mann-Whitney-test for non-normally distributed, continuous variables. For categorical variables, the Chi-square test or Fisher’s exact test (if appropriate) were used. Results were stated as mean and standard deviation in case of normally distributed data and as median, 25^th^ and 75^th^ percentile for non-normally distributed data. Correlations were performed according to Pearson (continuous variables) or Spearman (non-continuous variables). To evaluate the effect on the clinical status while adjusting for confounders and interactions between variables, a multivariate analysis was conducted. Two logistic regression models, one for progressive disease at any point in time and one for cured disease at baseline were designed. With respect to the sample size, a maximum of ten variables was included into the model to prevent over-fitting. Before the final model was established, a screening for multicollinearity was conducted, excluding variables with a variance inflation factor (VIF) of 10.0 or higher. Prior to the multivariate analysis, multiple imputation was performed to adjust for missing dependant variables. As many auxiliary variables and cases as possible were added to the imputation process to produce an accurate dataset. The number of data sets generated was determined using the formula suggested by Newgard and Haukoos [37], aiming for a relative efficiency of at least 95%, which is considered a high rate. Results were considered significant with a *p*-value (probability to make a type I error) smaller than 5%.

The statistical package JMP 14.2.0 was used for analyses of chemokine serum levels and the cellular production of cytokines and chemokines in patients with progressive, stable and cured AE. Significant differences between groups were determined with Tukey’s test after logarithmic transformation to stabilize the variance of data, either of the brut production (chemokine concentration in supernatants), or the net production (brut production minus chemokine concentration of resting PBMC (baseline)). The net productions were calculated according to the following formula: [production index = (brut production + 1)/(basline production + 1)]. We added one to the numerator and the denominator because of zero values in baseline and brut productions. For multiple comparisons, and to avoid type I errors, differences between infection groups were analyzed by the Tukey test. Bonferroni adjustment was applied, P values below p<0.001 are considered.

## Results

### Timetable of follow-up of clinical examinations of AE patients

AE patients’ follow-up visits were conducted at different points in time as displayed in Table 1. Of 63 patients at baseline, 12 patients could be follow-up for a mean of 11.3 years.

**Table 1:** Time table of follow-up visits of AE patients. Vx = follow-up visit, N = number of cases that adhered to follow-up visit, Δ_tVx-tV0_ = difference between baseline and follow-up visit.

V_x_	N	Δ_tVx-tV0_
V_0_	63	-
V_1_	40	5.3 ± 1.9 months
V_2_	43	11.7 ± 2.1 months
V_3_	13	23.2 ± 5.5 months = 1.5 ± 0.7 years
V_4_	52	65.4 ± 8.8 months = 5.1 ± 0.9 years
V_5_	12	141.3 ± 8.9 months = 11.3 ± 0.7 years

### *Echinococcus* IgG indirect hemagglutinin test (IHA) results in AE patients over time

Table 2 demonstrates the different levels of IgG IHA at different points in time. As many patients did not show elevated IgG IHA levels (coded as ‘0’) medians were therefore ‘0’. To display more subtle differences between groups, the respective mean and standard deviation were used as auxiliary variables in spite of the non-normal distribution A Wilcoxon test was performed to conduct group wise comparisons between baseline and follow-up visits. Only after a mean of 5 years (Visit 4) there was slight evidence (*p* = 0.05) that there was a significant decrease of IgG levels over time. The results displayed in Table 2 indicate a decrease in IgG IHA levels compared to baseline (*p* = 0.05) with a moderate Cohen’s effect size (*r* = 0.27). This difference became clearer comparing IgG IHA levels after six months and five years (*Z* = -2.092, N = 23, *p* = 0.036) with a strong Cohen’s effect size of 0.44.

**Table 2:** Level of IgG IHA over time and difference of respective levels compared to baseline. Vx = follow-up visit with V0 = baseline, N = number, M = mean, SD = standard deviation; MD = median, P_25_/P_75 =_ 25^th^ & 75^th^ percentile, Min. = minimum, Max. = maximum, *Z* = Wilcoxon’s Z-Value, *p*-value = significance level with * considered significant.

V_x_	N	M	SD	MD, 1:xx	P_25_-P_75_	*Min*	*Max*	*Z*	*p*
V_0_	63	98.1	293.95	0.0	0.0–81.5	0.0	2048.0	-	-
V_1_	37	111.7	344.20	0.0	0.0–81.5	0.0	2048.0	-0.566	0.572
V_2_	43	87.1	203.27	0.0	0.0–32.0	0.0	1024.0	-0.462	0.644
V_3_	12	125.33	291.84	32.0	0.0–64.0	0.0	1024.0	-0.954	0.340
V_4_	51		82.88	0.0	0.0–0.0	0.0	512.0	-1.959	0.050*
V_5_	12	164.92	566.11	0.0	0.0–16.0	0.0	2048.0	0.000	1.000

### *E*. *multilocularis* Em2+ antigen specific IgG responses in AE patients over time

Antibodies against Em2+ were measured as dichotomous variable. At baseline (V_0_), these antibodies were detected in 67 of 100 patients. Regarding the follow-up visits, a one-sided binominal test was conducted to evaluate if the observed distribution of detectable Em2+ antibodies differed from the expected distribution (0.67) at the different points in time. The results are displayed in Table 3. Overall, the percentage of patients with antibodies against Em2+ was lower upon follow-up visits compared to baseline. At V_1_ and V_2_ the observed and expected distribution differed significantly. At V_3_ and V_5_ sample size was too small and at V_4_ the effect size was insufficient to yield significant results. Detectable antibodies against Em2+ correlated with a higher level of Echinococcus IgG IHA (*s* = 0.343, *p* = 0.001).

**Table 3:** Number and percentage of AE patients with detectable Em2+ antibodies at different visits and significance level in binominal test. Vx = follow-up visit with V0 = baseline, N(%) = total number (percentage of patients) with detectable Em2+, *p*-value = significance level with * considered significant.

V_x_	N(%)	*p*
V_0_	67 (67.0%)	-
V_1_	18 (50.0%)	0.040*
V_2_	22 (51.2%)	0.023*
V_3_	6 (50.0%)	0.171
V_4_	31 (60.8%)	0.212
V_5_	7 (53.8%)	0.233

### *E*. *multilocularis* antigen specific IgG4 responsiveness in AE patients over time

Table 4 demonstrates the IgG4 responses (OD) to larval extract of *E*. *multilocularis* (EmAg) over the course of study. Paired t-tests were conducted with the subgroups V_0_, V_1_ and V_4_, e.g. at baseline, after around five months and five years. The sample size in the remaining subgroups did not meet criteria of a power level of at least 0.8 and were hence not included in further analysis. Compared to baseline, levels of IgG4 EmAg decreased over time. This effect was significantly after around five years (p = 0.028) with a moderate Cohen’s effect size (*r* = 0.37). The decrease between V_1_ and V_4_ was not significant (*t*(21) = 0.975, *p* = 0.341).

**Table 4:** Reactivity (indicated as optical densities OD) of IgG4 to larval extract of E. multilocularis (EmAg) over time and difference of respective levels compared to baseline. Vx = follow-up visit with V0 = baseline, N = number, M = mean, MD = median, SD = standard deviation; Min. = minimum, Max. = maximum, *t* = difference in units of standard error, df = degrees of freedom, *p*-value = significance level with * considered significant.

Vx	N	M	MD	SD	Min	Max	*t(df)*	*p*
V0	84	0.560	0.626	0.337	0.054	1.2	*-*	*-*
V1	36	0.558	0.625	0.283	0.064	0.978	*0*.*896 (34)*	*0*.*376*
V2	9	0.419	0.429	0.280	0.102	0.919	*-*	*-*
V3	1	1.105	1.105	-	1.105	1.105	*-*	*-*
V4	43	0.373	0.226	0.345	0.049	1.211	*3*.*298 (33)*	*0*.002*
V5	7	0.510	0.353	0.437	0.060	1.068	*-*	*-*

### Levels of total IgE and *E*. *multilocularis*-specific IgE in AE patients over time

Table 5 shows the level of total and *E*. *multilocularis*-specific levels of IgE at different points in time. A paired t-test was conducted to assess differences between baseline values and the IgE levels at respective points in time. Since levels of specific IgE were measured either on V_2_ or V_3_ in most cases, these subgroups were merged for the following analysis. Hence, the numbers in V_2/3_ represent specific IgE levels around one to two years after baseline measurements. At V_5_, no measurements of specific IgE levels were conducted. While no significant alteration in total IgE can be found over the course of disease, mean and median values have a decreasing tendency over time. There is a significant decrease in specific IgE after one to two years with a moderate Cohen’s effect size (0.31). There was a positive correlation between *Echinococcus* IgG IHA and total IgE (s = 0.549, p < 0.001).

**Table 5:** Levels of total and *E*.*multilocularis*-specific IgE in kU/l over time and difference of respective levels compared to baseline. Vx = follow-up visit with V0 = baseline, N = number, M = mean, MD = median, SD = standard deviation; Min. = minimum, Max. = maximum, *t* = difference in units of standard error, df = degrees of freedom, *p*-value = significance level with * considered significant.

**V**_**x**_	**N**	**M**	**MD**	**SD**	**Min**	**Max**	***t*(df)**	***p***
**Levels of total IgE in kU/l**								
V_0_	106	474.0	77.5	1,283.5	1.0	1,1150.0	-	-
V_1_	40	377.2	51.4	875.4	3.3	4,765.0	-0.512 (38)	0.612
V_2_	43	330.1	49.9	754.5	0.0	4,373.0	-0.216 (41)	0.830
V_3_	13	136.0	84.4	218.9	9.5	841.0	-0.475 (12)	0.643
V_4_	51	296.3	63.3	926.2	1.1	6,286.0	-0.024 (48)	0.981
V_5_	12	407.6	63.6	792.7	2.5	2,363.0	-1.049 (10)	0.319
**Levels of specific IgE in kU/l**								
V_0_	76	2.1	0.5	4.5	0.00	24.1	-	-
V_1_	19	0.00	0.00	0.00	0.00	0.0	1.000 (18)	0.331
V_2/3_	48	1.4		2.7	0.00	15.0	2.127 (47)	0.035*
V_4_	36	1.6	0.4	3.0	0.00	12.7	0.050 (34)	0.961

### Cytokine and chemokine serum levels in AE patients over time

Cytokine levels are displayed in Table 6. Log transformation of values was performed prior to further analysis. For IL-8, IL-9, CCL18 and CCL20, no significant changes between baseline and the follow-up visits were found (paired *t*-test). Levels of IL-10 were significantly lower at V_4_ (*t*(49) = 4.078, *p* < 0.001) and V_5_ (*t*(11) = 4.052, *p* = 0.002) comparing to baseline, reflecting a significant decrease after five and ten years. The further decrease of IL-10 levels between five and ten years after baseline was again significant (*t*(6) = 2.554, *p* = 0.043). The decrease in CCL13 was highly significant after 5 years (*t*(49) = 5.151, *p* < 0.000) and ten years (*t*(11) = 3.278, *p* = 0.007). Compared to baseline, levels of CCL17 were significantly lower after five years (*t*(48) = 2.915, *p* = 0.005) and ten years (*t*(12) = 3.025, *p* = 0.011) compared to baseline. Levels seem to increase again between five and ten years after baseline (*t*(7) = -2.403, *p* = 0.047), however, the sample size was small and the difference barely significant.

**Table 6:** Levels of cytokines and chemokines over time. Cytokines IL-8(CXCL8), IL-9, IL-10 and chemokines MCP-4(CCL13), TARC(CCL17), PARC(CCL18) and LARC(CCL20) were quantified in pg/ml in AE patients over time. Vx = follow-up visit with V0 = baseline, N = number, M = mean, SD = standard deviation; Min. = minimum, Max. = maximum.

V_x_	**N**	**IL-8(CXCL8)**					**IL-9**				
		M	MD	SD	Min	Max	M	MD	SD	Min	Max
V_0_	108	175.6	0.0	1,558.1	0.0	1,6191.0	44.8	0.0	128.3	0.0	930.4
V_1_	36	16,4	0.0	69.6	0.0	417.0	73.1	0.0	135.9	0.0	502.4
V_2_	9	89.7	0.0	269.0	0.0	807.0	180.4	0.0	328.3	0.0	970.4
V_3_	1	0.0	0.0	-	0.0	0.00	0.0	0.0	-	0.0	0.00
V_4_	50	24,3	0.0	137.2	0.0	966.0	73.4	0.0	319.4	0.0	1,902.7
V_5_	12	2,551.6	0.0	8,272.6	0.0	28,767.0	0.0	0.0	0.00	0.0	0.00
V_x_	**N**	**IL-10**									
		M	MD	SD	Min	Max					
V_0_	108	483.8	108.0	1,334.6	0.0	9,778.0					
V_1_	36	415.7	103.0	804.3	0.0	3,391.0					
V_2_	9	1,277.4	86.0	3,190.3	0.0	9,745.0					
V_3_	1	7.0	7.0	-	7.0	7.0					
V_4_	50	359.4	31.50	1,339.3	0.0	7,586.0					
V_5_	12	125.3	0.0	405.48	0.0	1,411.0					
**V**_**x**_	N	**MCP-4(CCL13)**					**TARC(CCL17)**				
		M	MD	SD	Min	Max	M	MD	SD	Min	Max
V_0_	108	333.6	176.5	442.9	6.0	2,757.0	480.3	205.0	812.3	0.0	4,825.0
V_1_	36	364.5	321.5	310.9	25.0	2,665.0	562.0	332.0	705.1	0.0	3,571.0
V_2_	9	531.9	265.0	857.4	55.0	766.0	527.3	167.0	1,005.9	0.0	3,303.0
V_3_	1	237.0	237.0	-	237.0	237.0	448.0	448.0	-	448.0	448.0
V_4_	50	117.0	66.5	143.1	0.0	812.0	114.7	17.0	277.0	0.0	1,468.0
V_5_	12	227.8	114.0	400.9	18.0	1,468.0	388.6	185.0	528.6	0.0	1,523.0
**V**_**x**_	**N**	**PARC(CCL18)**					**LARC(CCL20)**				
		M	MD	SD	Min	Max	M	MD	SD	Min	Max
V_0_	108	119,810.1	107,982.0	69,413.9	0.0	346,894.0	221.9	0.0	841.5	0.0	5,707.0
V_1_	36	122,144.0	107,982.0	81,536.8	0.0	347,700.0	180.1	0.0	471.3	0.0	2,353.0
V_2_	9	123,083.1	132,974.0	84,472.3	9,716.0	294,040.0	682.4	0.0	1,925.5	0.0	6,128.0
V_3_	1	139,982.0	139,982.0	-	139,982.0	139,982.0	0.0	0.0	-	0.0	0.0
V_4_	50	116,577.6	119,184.0	83,744.7	0.0	322,289.0	48.6	0.0	215.2	0.0	1,160.0
V_5_	12	116,071.0	119,897.0	69,963.7	0.0	230,965.0	91.8	0.0	330.9	0.0	1,193.0

### *E*. *multilocularis* patients with progressive, stable and cured AE

For the analyses of EmAg specific IgG isotype responses, 217 blood samples were collected from AE patients (Table 7). The AE patients were staged into three groups: those with progressive AE, with stable and cured AE. At year 1 of the study, the proportion of patients staged with progressive AE disease was 21.8%, in year 5 it reduced to 7% and in year 10 to 8.5%. The proportion of patients with cured AE increased from year 1 with 17.8% to 24.6% and 28.8% in year 5 and year 10, respectively. Patients with stable AE comprised the largest group (n = 137) and with 60%, 62% and 68% the proportion of AE stable remained similar at the three time points of study (Table 7).

**Table 7:** Alveolar echinococcus (AE) patients staged into groups with progressive, with stable and cured disease. The mean age with minimum and maximum ist indicated in brackets. The average age was 58.7 years. The two youngest patients were 19, the oldest patient 106 years old. One third of the patients were between 61 and 75 years old at the time of blood collection, and 51% percent of the patients were female. The youngest age group (0-30years) was the smallest with 8%, and the higher age groups of 31-45y, 46-60y, 61-75y and 76+y represented 16%, 24%, 36% and 16% of all AE patients, respectively.

	AE Progressive	AE Stable	AE Cured	N
Year 1	22 (22%)	61 (60%)	18 (18%)	101
	[63,8y (56;70)]	[53,9y (23;76)]	[59,1y (26; 106)	
Year 5	4 (7%)	39 (68%)	14 (25%)	57
	[65y (65; 65)]	[48,4y (28; 74)]	51,7y [30; 75]	
Year 10	5 (8%)	37 (63%)	17 (29%)	59
	[54,2y (19;76)]	[61,5 (32; 89]	59,1y [30; 80)]	
**Total**	**31 (14%)**	**137 (63)**	**49 (23%)**	**217**

### *E*. *multilocularis* antigen (EmAg) specific IgG isotype responses

**Fig 1:**
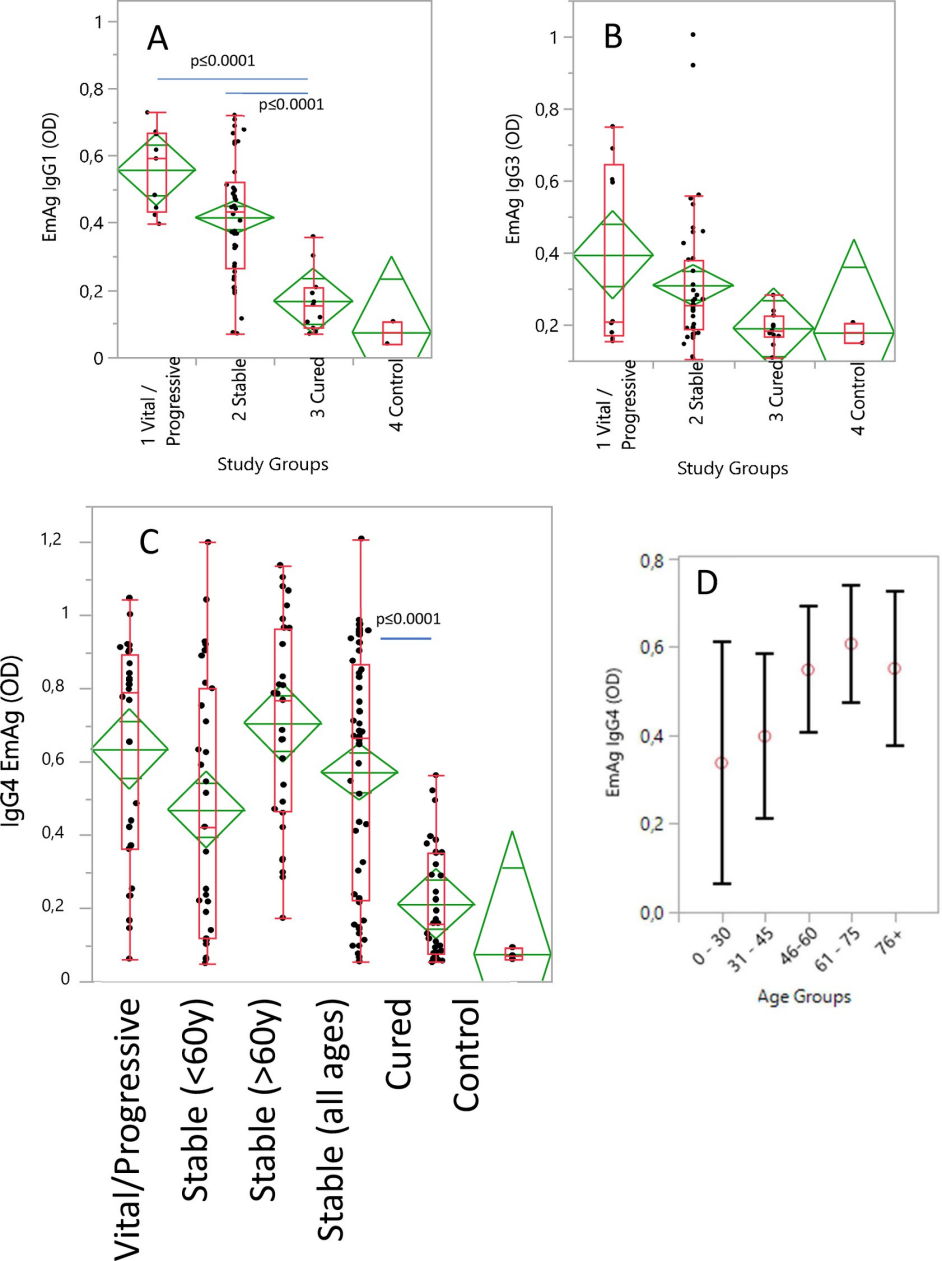
The *E*. *multilocularis* antigen-specific IgG reactivity (OD) in alveolar echinococcosis (AE) patients and infection-free controls. The AE patients were staged into those with progressive AE, with stable (<60y), stable (≥60) and cured AE. For the determination of age dependent EmAg specific IgG4 responses, the patients were grouped in five age groups. Blood samples from AE patients were collected at three time points in 2005 (year 1) in 2010 (year 5) and in 2016 (year10). Controls were echinococcosis infection-free healthy blood donors. In **(A)** the EmAg specific IgG1, in **(B)** the EmAg specific IgG3 and in **(C)** the EmAg specific IgG4 responses are shown as mean optical densities (OD) with the 95% confidence intervals for the means (diamonds). The data presented in box plots show the median OD per group with the 25% and 75% quartiles and the 1,5x of the interquartile range with outlier as individual points. In **(D)** the IgG4 reactivity in AE patients to EmAg is shown as mean OD per age group with the 95% confidence intervals.

The EmAg specific IgG responses were highest with progressive, lower in stable and lowest in cured AE patients (Fig 1). The EmAg-specific IgG1 responses were significantly higher with progressive (p = 0.0001) and stable (all ages) (p≤0.0001) than in cured AE patients (Fig 1, Part A). Similarly, the EmAg-specific IgG3 responses were highest with progressive, lower with stable (all ages) and lowest in cured AE patients, without significant differences between the study groups (Fig 1, Part B; Tukey Kramer Test). The EmAg specific IgG4 responses in progressive and stable (≥60y) AE patients were significantly higher (p<0.0001) than in cured AE patients (Fig 1, Part C). In cured AE patients, IgG4 responses were significantly lower (p<0.0001) than at the progressive, stable (all age groups) and stable (≥60y) stage of disease (Fig 1, Part C). In infection-free controls, IgG4 reactivity was slightly lower than in cured AE patients.

For the determination of age dependent EmAg specific IgG4 responses, the patients were grouped in five age groups (Fig 1, Part D). The youngest age group of patients up to 30 years showed the lowest and the 61–75 years old patients the highest IgG4 reactivity. Up to the age of 61–74 years the mean OD values increased steadily, but in the oldest patients (76+ years) slightly lower IgG4 responses to EmAg were measured. Significantly different EmAg-specific IgG4 responses (p = 0.041) were found between the age groups of 0–30 and 61–75 years. In younger stable AE patients (≤60y) the EmAg-specific IgG4 responses were measured lower than in older stable (≥60y) AE patients (p = 0.021).

### Chemokine serum levels in patients with progressive, stable and cured AE

Pro-inflammatory IL-8(CXCL8), regulatory IL-10 and TH-2 type IL-9 cytokines were measured in serum samples of AE patients and infection-free controls using specific ELISA (Table 8). The neutrophil-activating protein IL-8 concentrations were lowest in progressive and stable AE patients, enhanced in controls, and highest in cured AE patients, and differences did not attain significance. The Th2-type cytokine IL-9 levels were highest in stable (≤60y) AE patients, slightly lower in progressive AE, older stable (≥60y) and cured AE cases and at similar levels as measured in infection-free controls (Table 8). The regulatory cytokine IL-10 serum levels were highest in controls and younger (≤60y) AE patients, and less in progressive stable (≥60y) and cured AE case.

**Table 8:** Serum concentrations of cytokines and chemokines. Cytokines IL-8, IL-9 and IL-10 and chemokines MCP-4(CCL13), TARC(CCL17), PARC(CCL-18), LARC(CCL20) concentrations in pg/ml (mean [min, max]) were quantified by specific sandwich ELISA. The alveolar echinococcosis (AE) patients were staged in 4 groups with progressive, stable with equal or younger than 60 years (≤60y), stable older than 60 years (>60), and patients with cured AE. Control individuals were *E*. *multilocularis* infection-free. For IL-8: *Tukey-Kramer Test: p = 0.04 Cured vs. Control and vs. Stable and vs. Vital/Progressive; For LARC(CCL20) **Tukey-Kramer Test: p = 0.001 for Cured vs. Control.

AE Study Group	n	IL-8(CXCL8) (pg/ml) [min;max]	IL-9	IL-10	MCP-4 (CCL13)	TARC (CCL17)	PARC (CCL18) (ng/ml)	LARC (CCL20)
**Progressive**	31	2 [0; 29]	66 [0; 1,186]	153 [0; 1991]	226 [15; 1391]	545 [0; 2115]	100 [0; 200]	79 [0; 1560]
**Stable (<60y)**	35	41 [0; 966]	98 [0; 1903]	699 [0; 7586]	307 [6; 2529]	357 [0; 2558]	97 [0; 200]	135 [0; 1812]
**Stable (≥60)**	36	37 [0; 417]	31 [0; 378]	311 [0; 3606]	266 [18; 1117]	284 [0; 2641]	100 [0; 300]	149 [0; 2353]
**Cured**	49	969 [0; 28768]*	23 [0; 274]	223 [0; 2698]	224 [0; 1468]	215 [0; 1523]	100 [0; 200]	65 [0; 1193]
**Control**	35	318 [0; 2015]	46 [0; 775]	709 [0; 8572]	515 [101; 1978]	687 [1; 2785]	200 [0; 500]	641 [0; 4110]**

The CC chemokines TARC(CCL17), PARC(CCL18) and LARC(CCL20) were measured in serum samples of AE patients and infection-free controls (Table 8). Tissue and activation regulated chemokine CCL17(TARC) levels in sera steadily diminished from progressive to stable and cured AE patients, while being highest in controls. Pulmonary and activation regulated chemokine CCL18(PARC) serum concentrations were equally high (≥10.000pg/ml) in AE patient groups and controls. Liver and activation regulated chemokine CCL20(LARC) was similarly low in all patient groups, but significantly higher in controls than in cured AE patients.

### *E*. *multilocularis* antigen-induced cellular cytokine and chemokine production

**Fig 2:**
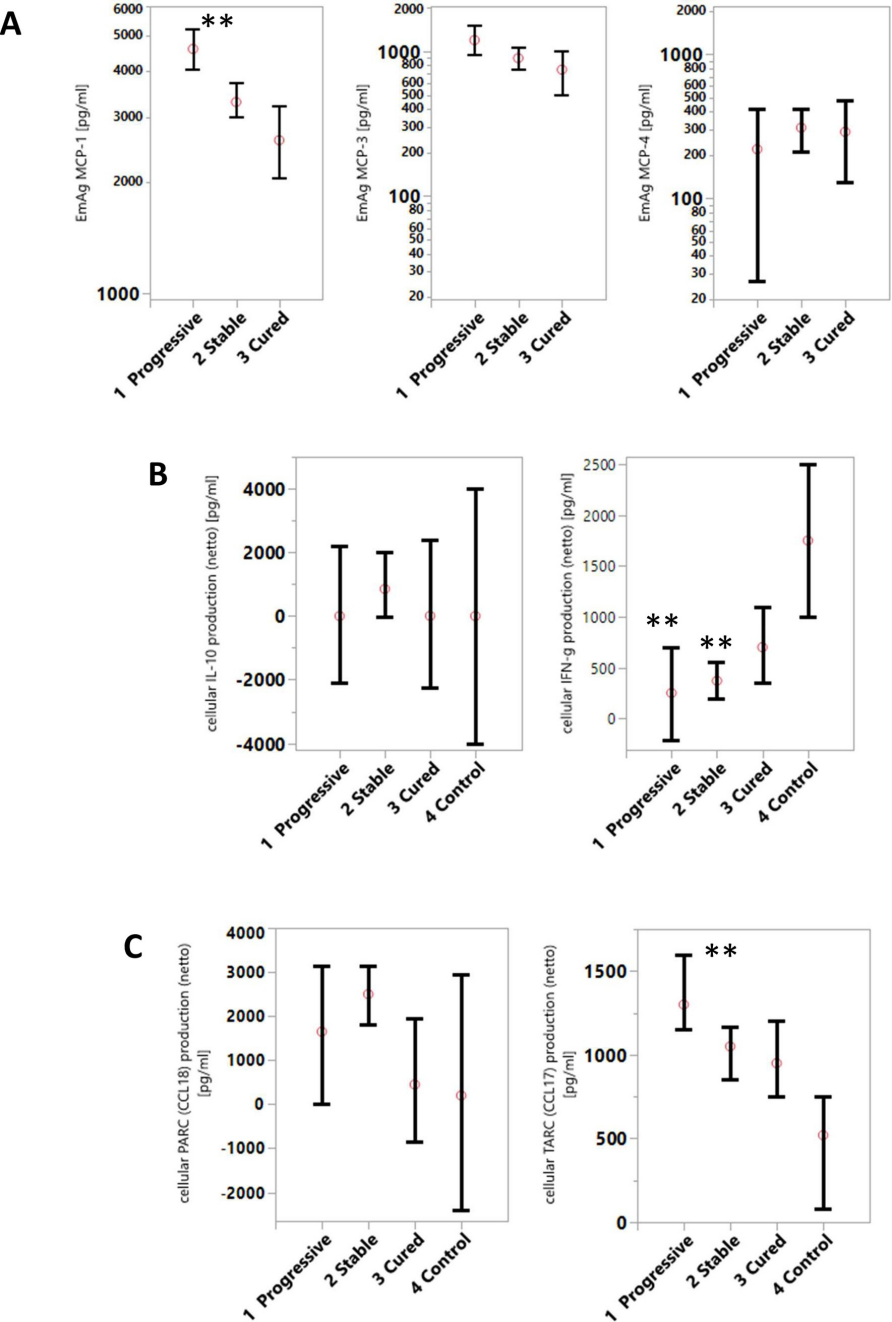
The *E*. *multilocularis* antigen inducible cellular production by PBMC of chemokines and cytokines. In (**A)** MCP-1(CCL2), MCP-3(CCL7), MCP-4(CCL13), in (**B)** cytokines IL-10 and IFN-γ and in (**C)** chemokines CCL17(TARC) and CCL18(PARC) were investigated in patients with progressive, stable, and cured AE, and in *E*. *multilocularis* infection-free controls. Freshly isolated and *in vitro* cultured PBMC (2.5x10^6^/ml) were stimulated with *E*. *multilocularis* antigen (EmAg, 12 μg/ml) or remained without stimulation (baseline) for 48 hours. Cytokines and chemokines secreted into cell culture supernatants were quantified with specific ELISA. The concentrations are indicated as mean (net) amounts in pg/ml (with the 5% upper and 95% lower confidence interval) of cytokine or chemokine released from stimulated PBMC minus the cellular production of unstimulated (baseline) PBMC. MCP-1(CCL2), MCP-3(CCL7) and MCP-4(CCL13) production by PBMC was quantified in AE patients with progressive (n = 7) stable (n = 29) and cured disease (n = 10) disease. For IL-10, IFN-γ and PARC(CCL18) and TARC(CCL17) production was investigated in AE patients with progressive (n = 19), stable (n = 27) and cured disease (n = 14), and in *E*. *multilocularis* infection-free controls (n = 6).** p<0.01 versus cured or control.

To study the chemokine (MCP-1, MCP-3, MCP-4, PARC, and TARC) and cytokine (IL-10, IFN-γ) production induced by *E*. *multilocularis* antigen (EmAg), PBMCs from AE patients were stimulated and the release of cytokines and chemokines into cell culture supernatants was quantified by specific ELISA (Fig 2). The monocyte chemoattractant MCP-1 production by PBMC was significantly higher in patients with progressive than in stable (p = 0.0006) and cured (p = 0.0002) patients (Fig 2, Part A). MCP-3 cellular production was slightly higher with progressive than stable AE patients (p = 0.03). No differences were observed for MCP-4 between the AE patient groups. The EmAg inducible IL-10 release was highest in patients with stable AE, and less in patients with vital/progressive or cured AE, and differences did not attain significance. The EmAg-stimulated cellular release of IFN-γ by PBMC was significantly lower in patients with progressive and stable AE (p<0.01) than in infection-free controls (Fig 2, Part B). The activation regulated chemokines TARC(CCL17), and PARC(CCL18) were produced lowest by PBMC from cured AE patients and infection-free controls (for TARC p<0.01 versus controls) (Fig 2, Part C).

### Correlations of cytokines and chemokines

#### Paired sample correlations of parameters in AE patients

The EmAg IgG4 responses correlated positive (R = 0.425, p<0.0001) with an enhancing AE disease severity (stage 1–4). The EmAg-specific IgG4 reactivity associated weakly with age (R = 0.222; p = 0.056) (Table 9) and the patients’ age did not correlate with the AE disease severity stages. Significant positive cytokine and chemokine correlations were observed in AE patients for IL-8 and IL-9 with TARC(CCL17) and LARC(CCL20), and for IL-10 with IL-8, IL-9, MCP-4(CCL13), TARC, LARC and PARC (for all p<0.001). Negatively correlated were cytokines ÍL-8, IL-9, IL-10, and chemokines MCP-4, TARC and LARC with the study years. Solely TARC correlated positive with the EmAg-specific IgG4 responses (R = 0.24, p = 0.0011). With the AE disease status negatively correlated were IL-8 (R = -0.462, p<0.0001), IL-10 (R = -0.157, p = 0.013), MCP-4 (R = -0.142, p = 0.024) and LARC (R = -0.262, p<0.0001).

**Table 9:** Paired Correlations of serum levels of cytokines and chemokines (in pg/ml) in AE patients, with age (in years), EmAg-specific IgG4 responses (OD values), stage of AE disease (4:progressive, 3:stable, 2:cured; 1:control) (study groups: n = 217 AE patients, n = 30 infection-free controls) and year of sample collection (year 1, 5, 10) are shown.

Variable 1	Variable 2	Corre lation	pairs N	lower 95% CI	upper 95% CI	p
AE Disease Status (1–4)	IgG4 EmAg (OD)	0,4259	185	0,3001	0,5371	<,0001
AE Disease Status (1–4)	Study Year (1,5,10)	-0,1738	217	-0,3001	-0,0416	0,0103
Age (y)	IgG4 EmAg (OD)	0,2116	82	-0,0056	0,4098	0,0563
Age (y)	AE Disease Status (1–4)	-0,0962	105	-0,2827	0,0972	0,3288
Age (y)	Study Year (1,5,10)	0,0548	105	-0,1383	0,2439	0,5787
IL-8 [pg/ml]	LARC(CCL20)	0,5431	247	0,4487	0,6255	<,0001
IL-8	AE Disease Status (1–4)	-0,4629	247	-0,5556	-0,3588	<,0001
IL-8	TARC(CCL17)	0,3602	245	0,246	0,4645	<,0001
IL-8	Study Year (1,5,10)	0,1485	217	0,0156	0,2762	0,0288
IL-9 [pg/ml]	LARC(CCL20)	0,4761	247	0,3735	0,5672	<,0001
IL-9	MCP-4(CCL13)	0,42	247	0,3115	0,5177	<,0001
IL-9	Study Year (1,5,10)	-0,3336	217	-0,4469	-0,2097	<,0001
IL-9	IL-8	0,2545	247	0,1339	0,3676	<,0001
IL-9	TARC(CCL17)	0,2483	245	0,1269	0,3623	<,0001
IL-10 [pg/ml]	IL-8	0,3795	247	0,2673	0,4815	<,0001
IL-10	IL-9	0,4841	247	0,3824	0,5742	<,0001
IL-10	MCP-4(CCL13)	0,6874	247	0,6154	0,748	<,0001
IL-10	PARC(CCL18)	0,2226	247	0,1005	0,338	0,0004
IL-10	TARC(CCL17)	0,2844	245	0,165	0,3957	<,0001
IL-10	LARC(CCL20)	0,5536	247	0,4607	0,6346	<,0001
IL-10	Study Year (1,5,10)	-0,2244	217	-0,3472	-0,094	0,0009
IL-10	AE Disease Status (1–4)	-0,1576	247	-0,2769	-0,0334	0,0132
MCP-4(CCL13)	LARC(CCL20)	0,5592	247	0,467	0,6394	<,0001
MCP-4(CCL13)	PARC(CCL18)	0,1994	247	0,0765	0,3163	0,0016
MCP-4(CCL13)	TARC(CCL17)	0,439	245	0,3319	0,5349	<,0001
MCP-4(CCL13)	IL-8	0,3905	247	0,2793	0,4914	<,0001
MCP-4(CCL13)	Study Year (1,5,10)	-0,2536	217	-0,3741	-0,1246	0,0002
MCP-4(CCL13)	AE Disease Status (1–4)	-0,1435	247	-0,2636	-0,019	0,0241
TARC(CCL17)	LARC(CCL20)	0,3593	245	0,245	0,4638	<,0001
TARC(CCL17)	IgG4 EmAg (OD)	0,2414	180	0,0986	0,3745	0,0011
TARC(CCL17)	Study Year (1,5,10)	-0,1762	215	-0,3028	-0,0434	0,0096
LARC(CCL20)	AE Disease Status (1–4)	-0,2625	247	-0,3751	-0,1424	<,0001
LARC(CCL20)	Study Year (1,5,10)	-0,1382	217	-0,2665	-0,0051	0,042

#### Unpaired Pearson’s correlations of cytokine and chemokine levels in AE patients

Significant positive cytokine and chemokine correlations (Table 10) were observed in AE patients for IL-9 with IL-10, CCL13(MCP-4), CCL17(TARC) and CCL20(LARC), for IL-10 with CCL13, CCL17 and CCL20, for CCL13 with CCL17 and CCL20, and for CCL17 with CCL20 (for all p<0.001). Higher levels of EmAg specific IgG4 correlated with lower levels of CCL17 (*r* = -0.246, *p* = 0.025) and CCL18 (*r* = 0.428, *p* < 0.001).

**Table 10:** Pearson’s correlations of cytokine and chemokine levels observed in AE patients are shown. *r* = Pearson’s r, *p* = significance level with * considered significant.

Cytokine/Chemokine		IL-8	IL-9	IL-10	MCP-4 (CCL13)	TARC (CCL17)	PARC (CCL18)
**IL-9**	*r*	-0.01					
	*p*	0.994					
**IL-10**	*r*	0.013	0.645				
	*p*	0.893	<0.001*				
**MCP-4(CCL13)**	*r*	0.019	0.667	0.877			
	*p*	0.843	<0.001*	<0.001*			
**TARC(CCL17)**	*r*	0.037	0.425	0.777	0.723		
	*p*	0.706	<0.001*	<0.001*	<0.001*		
**PARC(CCL18)**	*r*	0.043	0.091	-0.110	0.001	0.010	
	*p*	0.660	0.354	0.256	0.993	0.921	
**LARC(CCL20)**	*r*	0.013	0.568	0.975	0.833	0.776	-0.151
	*p*	0.894	< 0.001*	<0.001*	<0.001*	<0.001*	0.121

### Associations with clinical status

Both serological markers, *Echinococcus* IgG IHA and antibodies against Em2+ differed between the respective clinical groups (Kruskal-Wallis-test, *Χ*^2^_IHA_(3) = 10.037, *p* = 0.018; *Χ*^2^_Em2_(3) = 19.341, *p* < 0.000). Mean ranks in both *Echinococcus* IgG IHA and Em2+ tended to increase from cured (31.0/30.6), stable without BMZ therapy (43.3/34.9), stable with BMZ therapy (55.5/55.7) to progressive disease (52.3/67.0).

IL-8 differed significantly between the clinical groups (one-way ANOVA) (*F*(3) = 3.070, *p* = 0.031). Cured patients showed significantly higher levels of IL-8 (M = 1619.1 pg/ml) than those stable with (M = 28.6 pg/ml) or without BMZ therapy (M = 29.1 pg/ml).

CCL17 levels tended to be lower in those cured (M = 103.60) compared to those stable without (M = 301.93) or with BMZ therapy (M = 557.63) or those with progressive disease (M = 544.33) (*F*(3) = 2.534, *p* = 0.061). Specific IgE levels tended to be higher in progressive disease (8.61 kU/l) compared to cured (2.01 kU/l) patients and those stable with (1.92 kU/l) and without BMZ therapy (1.52 kU/l), however, this result was not significant (*F*(3) = 2.336, *p* = 0.081).

### Multivariate analysis

To evaluate the effect on the clinical status while adjusting for confounders and interactions between variables, a multivariate analysis was conducted. Two models, one for progressive disease at any point in time and one for cured disease at baseline were designed. Since there was a strong correlation between cytokines, IL-8, IL-10 and CCL20 had to be removed from the model because of multicollinearity (variance inflation factor VIF > 10.0). Since specific IgE and total IgE as well as Em2+ and *Echinococcus* IgG IHA correlated strongly, the variable which led to highest increase in R^2^ was added to the model. Age and gender were added as effect modifiers. Prior to the multivariate analysis, multiple imputation was performed to adjust for missing dependant variables. Both models are displayed in Table 11.

Young age at first clinical presentation and high levels of specific IgE were significant risk factors to develop progressive disease. With every unit the specific IgE increases, the odds for progressive disease increase 1.3-fold. Similarly, the odds increase 1.1-fold with every decreasing year of age at baseline, e.g. at first presentation. High levels of CCL17 might also indicate progressive disease; however, the result missed the required significant level. Low or undetectable antibodies against Em2+ were significantly associated with being cured.

**Table 11:** Logistic regression models with variables associated with progressive or cured AE clinical status. OR = Odds, CI 95% = 95% confidence interval, *p* = significance level with * considered significant.

Outcome	Progressive AE			Cured AE		
Variable	OR	CI 95%	*p*	OR	CI 95%	*p*
Age	0.926	0.866–0.991	0.026*	0.955	0.903–1.011	0.112
Sex	0.871	0.122–6.212	0.891	0.323	0.064–1.632	0.172
Em2+ antibodies	n.a.	0.000 –n.a.	0.998	0.155	0.026–0.910	0.039*
IgG4 EmAg	0.996	0.963–1.031	0.827	1.000	0.998–1.002	0.995
WBC	0.989	0.976–1.002	0.107	1.002	0.999–1.004	0.198
Specific IgE	1.245	1.018–1.523	0.033*	0.562	0.150–2.101	0.386
IL-9	1.005	0.994–1.017	0.372	1.002	0.991–1.012	0.742
MCP-4(CCL13)	0.992	0.982–1.002	0.126	0.996	0.990–1.003	0.276
TARC(CCL17)	1.002	1.000–1.004	0.076	0.997	0.993–1.002	0.248
PARC(CCL18)	1.000	1.000–1.000	0.222	1.000	1.000–1.000	0.261

## Discussion

This study investigated the dynamics of *E*. *multilocularis* specific and systemic antibody and cytokine and chemokine levels, and, parasite-specific cellular responses during treatment of AE patients. The *E*. *multilocularis* antigen specific IgE and IgG reactivity and the systemic and parasite-specific cellular cytokine and chemokine responses were differentially expressed with the clinical state of disease. Most AE patients have experienced a state of disease with progressive parasite growth associated with high IgG4 responses [38]. IgG4 production is maintained by chronic helminth infection and supported by Th2-type cytokine responses [39,40]. In AE patients in whom larval lesions have regressed after partial surgical resection of parasite infested tissues or organ, remnants of the laminate layer of *E*. *multilocularis* may persist for many years and continue to stimulate specific IgG [41,42]. Our results demonstrate a rapid decrease in antibodies against Em2+, especially in cured patients; these antibodies remained negative, making them a significant predictor for cured AE. Previously observed, in patients after curative surgery, seroreversion in the Em2+ ELISA indicated successful resection of lesions in AE patients [41]. Antibodies directed against *E*. *multilocularis* crude antigens were detectable longer than antibodies directed against the recombinant Em18 antigen in all patient cohorts and stages with 95% sensitivity [42]. The IgG4 responses were similar in patients with vital/progressive and stable >60years disease, and only with cure, the IgG4 reactivity diminished significantly. Furthermore, in patients with stable disease, the IgG4 reactivity increased with age, indicating that inactive or residual cestode antigens will continue to stimulate IgG4. Em2 is a major carbohydrate antigen located on the laminated layer of metacestode larvae. Em2 inhibited polyclonal or parasite extract-induced splenocyte proliferation and triggered IgG3 responses, and together with the related carbohydrate rich Em492 both may contribute to immunosuppressive events and facilitate parasite survival [43,44]. With repeated longitudinal follow up of the *E*. *multilocularis*-specific antibody isotype responses, the objective would be to detect changes in AE disease activity that is not visible by imaging techniques. This requires long term biobanking of patients’ samples and repeated serological analyses. Parallel monitoring with imaging techniques and Em-specific IgE, IgG isotypes and Em2+ reactivity could therefore maximize diagnostic accuracy and inform treatment decisions.

Most AE patients were at stable AE and may remain such for life, while individuals in whom larval tissues regressed and parasite lesions calcified were considered immune competent to control AE progression [11]. Patients with Th1 dominant immune response were more likely to show a curative course of the disease, and Th2 responses were more frequently observed with progressive and vital larval growth [17]. In AE patients, the CD4+ T cell responses weakened over time, and the expansion of regulatory T cells (Treg) was considered an important factor in the immune evasion of *E*. *multilocularis* [12,14,24]. In experimentally *E*. *multilocularis* inoculated mice, the T cell responses were increasingly suppressed with parasite persistence, and depletion of FoxP3^+^ regulatory T cells as immunotherapy against either *E*. *multilocularis* oral infection with eggs or intraperitoneal injection of larvae, resulted in significantly smaller parasite lesions in the livers and lower parasite loads, respectively [45,46]. The balance between T cell response and parasite load determines the disease outcome of mice infected with different *E*. *multilocularis* inocula [47]; and a stronger expression of programmed cell-death receptors (PDCD1, 2B4 and LAG3) associated with “exhausted” parasite antigen induced T cell responses and an enhanced *E*. *multilocularis* metacestode growth at the late stages of infection [47]. In the present work, the Th1-type cytokine IFN-γ production was lower in EmAg-stimulated PBMC from patients with active and stable AE, and IFN-γ responsiveness enhanced with cured AE. Interestingly, therapy with IFN-γ bolus injection was applied to stop AE, and disease progression was not detected during the observed period of 18 months [48]. The injected IFN-γ did not change the TH-2 dominated immune response profile to *E*. *multilocularis*, but intracellular IL-10 was elevated [48].

In the sera of AE patients with progressive and stable AE, low concentrations of IL-8(CXCL8) were measured, and concentrations enhanced with cured AE and at year 10 of study. A substantial IL-8 production was observed when granulocytes of AE patients were stimulated with EmAg [35,36] while PBMC released only low amounts. The neutrophil-activating interleukin 8 (CXCL8) will trigger inflammatory responses, and angiogenesis and capillary tube formation are supported by IL-8(CXCL8) [49]. IL-8 is mainly active in primary innate responses and a lessened neutrophil-activating chemokine IL-8(CXCL8) cellular production may silence effector cell migration into *E*. *multilocularis*-infested tissues and favour parasite persistence. At year 10 of follow up, the serum levels of IL-8 enhanced, and increased angiogenic activity around *E*. *multilocularis* metacestode larvae may favour chemo attraction of effector granulocytes and trigger cytotoxic responses.

The T helper-type 9 subgroup (TH9) will produce IL 9 in large amounts, together with IL-4, IL-5 or IL-13, and IL-9 can suppress Th1-type cells and support intestinal parasite expulsion, as well as tissue renewal [27,50]. In patients with cystic echinococcosis, enhanced IL-9 concentrations and mRNA levels of TH9-type cells were detected when compared to controls [51]. In this study, IL-9 serum concentrations were similar in the AE patient groups, but at year 10 of study, levels of IL-9 and regulatory IL-10 diminished, suggesting a re-activation of Th1-type immune responses. The positive associations of IL-9 (p<0.001) with several studied cytokines and chemokines disclosed the gradual switch towards a Th1-type responsiveness.

IL-10 exerts anti-inflammatory effects during infections. It regulates and dampens immune responses against pathogens, thus circumventing inflammatory processes which may damage host tissues [52,53]. Regulatory T cell responses and IL-10 with AE are considered to play an important role in parasite tolerance [27] and an increased basal IL-10 secretion was found in patients with progressive AE [23]. Similarly, we detected lowest serum IL-10 with progressive AE, but IL-10 was similarly inducible in vital/progressive, stable, and cured AE cases, supporting persistent regulatory processes which may counteract inflammation and tissue damage. A spontaneous release of IL-10 by PBMC is considered a sign for a progressive course of the disease, and suppression of the innate immune response induced by IL-10 could promote parasite growth and survival. The positive associations of IL-10 (p<0.001, paired sample correlation) with all studied cytokines and chemokines disclosed its broad regulatory function on T-helper-cell subsets and highlighted IL-10 as a regulator of cellular responsiveness. The IL-10 production appears favourable for parasite persistence and T-helper-cell responses regulated by IL-10 and TGF-β may dampen proinflammatory Th1- and Th2-cells, thereby preventing pathogenic cellular hyperreactivity in AE patients.

The induction of the activation regulated chemokines TARC and PARC and the monocyte chemoattractant MCP-1 after stimulation of PBMCs with EmAg was lowest in stable and cured AE patients, which can be attributed to a suppressed or arrested proliferative growth of the metacestode larvae, possibly supported by long-term therapeutic interventions with benzimidazole. MCP-1 rather contributes to Th2 than to Th1 cytokine-mediated granulomatous inflammation and Th1 cell expansion [54]. The CC-chemokines MCP-3 and MCP-4 were similar in the patient groups making these chemokines less suitable as predictive disease marker [35]. The Th2-type chemokine TARC(CCL17) mediates chemotactic effects on macrophages, monocytes, and eosinophil and basophil granulocytes [55]. Levels of TARC are particularly elevated in inflammatory Th2-type responses with various skin diseases such as atopic dermatitis, bullous pemphigoid, or mycosis fungoides, especially during the acute phases [56]. In patently helminth-infested patients, the serum levels of TARC were strongly increased [57,58], and even higher in helminth and protozoa poly-parasite infections [59]. Levels of CCL17 decreased during treatment, especially in cured AE patients, and CCL17 might serve as a predictive or risk factor for progressive disease.

In AE patients, serum levels of the pulmonary and activation regulated chemokine PARC (100–500 ng/ml) and its cellular production was largely above the other cytokines and chemokines. PARC(CCL18) levels did not change with AE and were not correlated the other tested parameters. PARC(CCL18) is constitutively expressed in the lung and elevated concentrations are detected in pulmonary fibrosis [60,61].

The liver and activation regulated LARC(CCL20) exerts a chemotactic effect on immature dendritic cells (DC), effector/memory T-cells and B-cells, and Th17 cells [62]. LARC(CCL20) can induce migration of dendritic cells through the epithelial tissue to stimulate maturation and proliferation of T and B cells [63]. In *Trichuris muris* infection of mice, dendritic cell recruitment mediated by LARC(CCL20) and RANTES(CCL5) will determine immunity or chronic disease progression [64]. CCL20 contributes to the progression of many cancers, such as liver cancer, colon cancer, breast cancer, pancreatic cancer, and gastric cancer [65]. LARC(CCL20) and his sole receptor CCR6 will favour migration of Treg cells into a tumour microenvironment and disease progression in hepatocellular carcinoma [66]. Increased levels were detected in childhood with proliferative acute lymphoblastic leukemia [67] and LARC will promote cell metastatic spread in human colorectal malignancies [68]. The serum levels of LARC(CCL20) correlated positive with IL-9, IL-10, MCP-1(CCL13) and TARC(CCL17) disclosing their collective role on the expression of innate immune responses. Vital *E*. *multilocularis* larval tissues or residual not resolved parasite lesions may have stimulated their production, whether these chemokines affected larval growth remains uncertain, but they may serve as markers for AE staging.

In summary, the rapid decrease of antibodies against Em2+, especially in cured patients, makes them a significant predictor for cured AE. The positive relation of *E*. *multilocularis* specific IgG4 responses with chemokine TARC can indicate AE progression when both enhance over time. Inflammatory and regulatory cytokines and chemokines persisted in AE patients without an exclusive dominance of Th1-, Th2-, and Treg-type immune responsiveness. Enhanced levels of IL-8, IL-10, MCP4 and LARC may predict AE regression. Repeated biomarker surveys are advisable to evaluate progression or regression of AE during longitudinal follow up, and such analyses can support imaging techniques and improve staging of AE patients.
